# From Screen to Table: How Douyin Food Bloggers Stimulate and Convert Viewer Interests

**DOI:** 10.3390/bs14070602

**Published:** 2024-07-16

**Authors:** Ruoqing Guo, Ziqing Yang, Hao Gao

**Affiliations:** 1 School of Journalism and Communication, Nanjing Normal University, Nanjing 210097, China; 220202047@njnu.edu.cn (R.G.); 42396@njnu.edu.cn (H.G.); 2 School of Modern Circulation, Guangxi International Business Vocational College, Nanning 530007, China

**Keywords:** food exploration bloggers, source credibility, taste desire, taste awareness, visiting intention, SOR

## Abstract

In the era of social media, the influence of food exploration bloggers is increasingly apparent. Sharing their culinary experiences stimulates the audience’s interest in visiting and consuming food destinations. This paper seeks to understand how the characteristics of food exploration bloggers on the Douyin platform influence audience perceptions of food and locations and how these perceptions may relate to visiting intentions, using the stimulus–organism–response (SOR) model. A cross-sectional online survey analyzed responses from 437 individuals interested in food exploration videos on Douyin. The results indicate that source credibility is significantly associated with the stimulation of taste desires and the formation of taste awareness. The audience’s taste desire and taste awareness are positively linked to the intention to visit. This study contributes to the expansion of the SOR model’s application in digital media, underscoring the substantial role of social media in influencing audience consumption intentions. It highlights that as an effective communication tool, social media can significantly impact users’ behavioral responses and consumption decisions.

## 1. Introduction

Social media platforms have become crucial channels for entertainment and information acquisition. Reports indicate that China currently has 1.03 billion social media users, accounting for 72% of its population [1]. With the growing number of users, marketing strategies on social media have emerged and gradually become vital for brand communication and product sales. Studies show that social media significantly increases sales, word-of-mouth, and profit and impacts brand value [2]. Additionally, the development of social media platforms benefits from establishing social networks among consumers, opinion leaders, and domain experts, thereby altering the dynamics of the e-market [3]. In this context, the role of social media influencers (SMIs) has become increasingly important.

Social media influencers (SMIs) play a crucial role in the marketing domain of social media platforms. Many brands realize that collaborating with SMIs allows for more direct engagement with their target audience [4]. Through such partnerships, brands can stimulate consumer recognition and effectively promote their products and services [5]. This influencer-based marketing strategy is called influencer marketing (IM) [6]. Douyin (TikTok Chinese version) has gained significant attention among all social media platforms due to its vast audience base. Occupying 75% of the social media user share in China, Douyin has become one of the most popular platforms in the country [7]. As a short video-sharing platform from ByteDance, Douyin hosts a multitude of content creators, some of whom have gained a substantial following and have become influential internet celebrities. Of the total, 43% of Douyin users state that their purchasing decisions are influenced by celebrity or influencer advertisements [7], highlighting the immense potential and importance of influencer marketing on Douyin.

In this context, although numerous studies have shown that social media influencers can influence consumer attitudes and behaviors [8,9,10], there has been limited research focusing specifically on influencers in particular domains on platforms like Douyin, such as food exploration. Food exploration bloggers on Douyin primarily create videos exploring local cuisines and restaurants, covering recipes, restaurant atmospheres, pricing, and detailed food appearance and taste descriptions. Some delve into cooking methods and the cultural stories behind the dishes. The high interest of Douyin users in cuisine and restaurants has given such bloggers a significant position on the platform. Some top food exploration bloggers have tens of millions of followers and up to hundreds of millions of likes. For example, the most followed food exploration blogger in this niche, *Te Bie Wu La La*, as of June 2024, has about 18.35 million followers and 230 million likes. His videos create a relaxed atmosphere, mainly showcasing his experiences with local delicacies across the country evaluating the taste of food and dining environments. Many tourists visit his recommended restaurants, and restaurant owners often use screenshots of his videos to attract more customers. 

Manaf confirms that the characteristics of food bloggers can influence the parasocial interactions between bloggers and viewers, affecting food tourists’ attitudes and behavioral tendencies [11]. Mainolfi et al. explored how food blogs influence readers’ emotional attachment to bloggers, increasing their willingness to visit food locations [12]. Current research primarily examines the emotional bonds between bloggers and audiences, leaving room to explore further how bloggers attract audience cognitive responses. Accordingly, this study aims to explore the influence mechanisms of food exploration bloggers on the Douyin platform and how these mechanisms translate into the actual behavior of the audience when visiting. The stimulus–organism–response (SOR) model proposed by Mehrabian and Russell provides the framework for this study [13]. Although this model has been widely applied and studied in marketing and consumer behavior [14,15], its application in food destination marketing is relatively scarce. Therefore, this study adopts the SOR model, taking the source credibility of bloggers as the stimulus variable, inspiring taste desire and forming taste awareness as the organism variables, and intention to visit as the response variable to address the following research questions:

1. How do food exploration bloggers on the Douyin platform influence consumer visiting behaviors?
2. How do different characteristics of food exploration bloggers on the Douyin platform affect audience perception of food destinations?
3. How is the audience’s perception of food and its locations transformed into visiting intentions?

This study aims to address gaps in current research concerning specific platforms and topics while examining how online content influences offline consumer decisions, providing new insights for brands and marketers. Additionally, this research enriches and expands the application of the stimulus–organism–response (SOR) model within digital media and social platforms, deepening our understanding of the SOR model in analyzing the impact of social media and offering empirical evidence and theoretical guidance for future studies in similar fields.

## 2. Theoretical Backdrop and Hypotheses Development

### 2.1. Social Media Influence

Early social media was primarily used for information sharing, entertainment, and interpersonal interaction [16,17,18]. In recent years, with the evolution of commerce, social media has transformed into a platform where users can socialize, create content, and make online purchases [19]. On the one hand, social media serves as a vast pool of information on consumer attitudes toward brands. On the other hand, it facilitates social interactions among consumers, leading to greater trust and directly influencing their purchasing behaviors [20]. The application of social media marketing extends beyond online to offline activities, especially in destination marketing. Social media has become crucial for promoting tourist destinations and attracting visitors. For instance, tourism agencies engage directly with potential tourists by sharing captivating images and videos of destinations on platforms like Instagram, Facebook, and TikTok, enhancing the attractiveness of these locations [21,22,23]. By sharing their travel experiences, social media influencers quickly attract followers and inspire their travel interests [24]. Social media has become essential for shaping perceptions, feelings, and experiences [25]. Promoting culinary destinations on social media has also caught some scholars’ attention. Wang noted that food blogs influence readers’ intentions to taste local food and beverages through electronic word of mouth [26]. Studies have also shown that food vlogs on Bilibili, a Chinese video platform, effectively spark interest in culinary destinations by showcasing authentic cuisine and culture, thus inspiring the audience’s willingness to visit these locations [27]. Douyin, as a digital media creation and dissemination tool, can effectively interact with target audiences and precisely push brand and product information to them [28]. The format of short or long videos on Douyin is highly suitable for displaying detailed visuals, fully presenting a location’s image from audio to visual [28]. Therefore, Douyin has become a significant focus of study in destination marketing. However, despite the widespread attention Douyin receives in promoting tourist destinations [29,30], research on culinary themes remains relatively insufficient. Considering the high popularity of culinary content on Douyin, conducting an in-depth study on marketing culinary locations on the platform is precious.

### 2.2. Stimulus–Organism–Response Paradigm

This study adopts the stimulus–organism–response (SOR) model as its primary analytical framework. Environmental psychologists originally proposed the model in the early 20th century to explain how environmental factors influence individual behavior. Mehrabian and Russell were early advocates of this model, emphasizing that external environmental stimuli affect an individual’s emotional state, triggering specific behavioral responses [13]. Later, Bitner extended the influence of environmental stimuli to the cognitive level, focusing on how the physical environment affects consumers’ and employees’ cognition and emotional responses [31]. Following digitalization and the emergence of social media, the model finds extensive use in online contexts to probe the effects of videos, images, and various forms of media content on the cognitive processes and behavioral responses of audiences [32,33]. Laroche et al. used the SOR model to analyze how animated images in an online retail environment enhance purchasing intentions by affecting consumers’ emotions and cognition, finding that animated images evoke greater pleasure compared to static images, leading to a more positive attitude towards the website and ultimately increasing purchase intentions [34]. Research has also applied the SOR model to study the impact of social media marketing, electronic reputation, and destination image on tourists’ visiting intentions, finding that destination image completely mediates the relationship between social media marketing, electronic reputation, and tourists’ visiting intentions [14]. Environmental psychology theories suggest that when viewers watch videos of food exploration bloggers on Douyin, they perceive the appearance and taste of the food, as well as the atmosphere of the restaurant, thereby forming specific cognitions about the restaurant [35,36]. These cognitions could trigger their gustatory desires, enhancing their willingness to visit. The SOR model provides a theoretical foundation for understanding and analyzing the transformation process from external stimuli to perceptual response to behavioral intention. In this study, we focus on how the characteristics of food exploration bloggers on Douyin (stimuli) influence viewers’ perceptions of the cuisine and culinary locations (organism) and subsequently affect their visiting intentions (response). This model’s structure helps us systematically deconstruct and evaluate how various blogger characteristic stimuli influence consumer decision-making paths.

### 2.3. Stimulus: Source Credibility

Food exploration videos on social media platforms (such as Douyin) are content that mainly showcases food bloggers exploring various restaurants and culinary spots. These bloggers gradually accumulate a fan base by sharing unique dining experiences and personal insights into different restaurants and cuisines, gaining corresponding influence and further affecting their followers’ behaviors [37]. When the food industry recognizes the influence of these food exploration bloggers and collaborates with them, it forms a practical marketing approach. These content creators have become the primary driving force behind influencer marketing in the food industry [38]. However, not all bloggers achieve this effect. In the digital age, consumers are increasingly sensitive to information, and the credibility of the information source becomes a key factor influencing their behavior [5].

This study adopts a four-dimensional conceptualization of source credibility to examine the credibility of food exploration bloggers, including trustworthiness, expertise, attractiveness, and similarity [39]. Trustworthiness and expertise are the two foundational source credibility factors [40]. Trustworthiness refers to the degree to which an information source is perceived as honest, sincere, or genuine [41]. When consumers believe a source is trustworthy, they typically also consider the information provided by the source as highly reliable [42]. Expertise involves the knowledge, ability, or qualification that the information source possesses, such as the products and services it advocates [43]. Individuals with high expertise are generally considered more persuasive [44]. DeBono et al’.s study also confirmed the direct impact of the source’s attractiveness on the effectiveness of information dissemination [45]. Attractiveness is not only reflected in the audience’s appreciation of the source’s physical appearance [46] but also includes the personal charisma exhibited by the communicator [47]. DeShields et al’.s research indicated that attractive food endorsers are more popular than unattractive ones and can inspire a stronger consumer purchase intention [48]. Additionally, similarity refers to the perceived likeness between the information source and the receiver, including shared characteristics between influencers and their fans [49]. Studies have shown that the higher the consumer’s perceived similarity to the endorser, the better the persuasive effect [50].

### 2.4. Organism: Inspiring Taste Desire and Forming Taste Awareness

Photos and films have been proven to help audiences feel the allure of landscapes and stimulate their desire to travel [51,52]. Similarly, Wang argued that food blogs, operating on a similar principle, can also stimulate gustatory desires and introduce the concepts of experiential attractiveness and resonance [26]. In this study, experiencing appeal refers to the level of psychological excitement experienced by viewers while watching food exploration videos, including sensations of excitement, curiosity, allure, and persuasion [26]. Research by Rust and Oliver showed that excitement and curiosity help to enhance the desire for experiential food tasting [53]. It is because people seek novelty when trying local foods, and this need for novelty drives the pursuit of exciting experiences. Generating empathy is defined as the extent to which the content of food exploration videos can elicit emotional and cognitive responses from viewers, such as the anticipation and desire to taste the food described by the blogger.

On the other hand, Wang suggests that food blogs, through the internet, provide information and choices related to cuisine and culinary destinations, aiding potential culinary tourists in forming a gustatory awareness in advance [26]. Its sub-variables include providing image, delivering knowledge, and presenting guides. In this study, providing image refers to how watching food exploration videos help individuals form an overall impression of a culinary location, involving food quality, service quality, dining environment, and comfort. Delivering knowledge is how food exploration videos help viewers understand local culture, such as local dining traditions and customs, table etiquette, and unfamiliar local dishes. Presenting guides refers to the capability of food exploration videos to offer valuable tips, such as healthy menu and food budget suggestions, contact information, addresses, maps, operating hours of restaurants, and tips to help viewers plan a personal visit to the establishment.

### 2.5. Response: Intention to Visit

Intention to visit is typically defined as consumers’ potential decision or inclination to participate in, experience, or visit a specific place or activity [54]. It is a critical factor in predicting consumer behavioral intentions and is seen as a bridge between actual behavior and psychological attitudes [55]. According to information processing theory [56], when consumers encounter certain stimuli, such as recommendations from a food exploration blogger, they undergo a series of reception, encoding, storage, and retrieval processes. These processes ultimately form cognitions and evaluations of food or restaurants. In this process, the characteristics of the blogger, especially their credibility, significantly influence the formation of the audience’s intention to visit. When consumers perceive an information source as highly credible, they are more likely to be influenced by the information and take action [57].

### 2.6. Hypotheses Development

Information sources act as a stimulus factor or a motive for image formation [58,59], crucial in influencing perception and evaluation formation. As primary information sources, food exploration bloggers create an immersive culinary experience for viewers through verbal descriptions and visual presentations. Research has pointed out that imagining eating pleasurable food will likely create a positive experience, increasing the desire for the imagined food [60]. On the other hand, by introducing the overall environment of culinary locations, food exploration bloggers also provide viewers with cues to construct the image of these places. Baloglu et al. indicated that images can be formed through information sources [58], and Beerli and Martin also emphasized that information sources affect the shaping of the overall image of destinations [61].

However, not all information will be received and adopted by the audience. Nadlifatin suggests that audiences need a trustworthy source of information [62]. A reliable source of destination information in tourism services increases positive perceptions of the destination’s image [63,64]. Credible travel information has been proven to significantly influence the choice of destinations [65]. Therefore, we hypothesize that, in the context of culinary experiences, the credibility of the information source is equally crucial for forming perceptions about food destinations.

**H1a.**  *Source credibility is positively correlated with inspiring taste desire.*

**H1b.**  *Source credibility is positively correlated with forming taste awareness.*

According to cognitive appraisal theory [66], individuals are likely to have a more positive intention to act when they perceive an activity as fulfilling their needs, such as curiosity and desire for exploration. Kozinets et al. also noted that digital technology can transform and stimulate consumers’ desires in modern consumer culture, especially regarding food and culinary experiences, translating online cravings for food into offline consumption experiences [67]. Moreover, today’s consumers need to engage with and consider many information sources before making purchase decisions [68]. Information about culinary locations obtained from food exploration bloggers’ videos can help viewers form a gustatory awareness of these places in advance.

Uncertainty reduction theory suggests that as consumers gain more information about food and restaurants, their uncertainty about these options decreases, potentially increasing their willingness to visit and dine [69]. This process indicates that the information provided by food exploration bloggers stimulates the audience’s gustatory desires and reduces uncertainty in the consumer decision-making process. Based on this, we hypothesize the following:

**H2a.**  *Inspiring taste desire is positively correlated with intention to visit.*

**H2b.**  *Forming taste awareness is positively correlated with intention to visit.*

**Figure 1:**
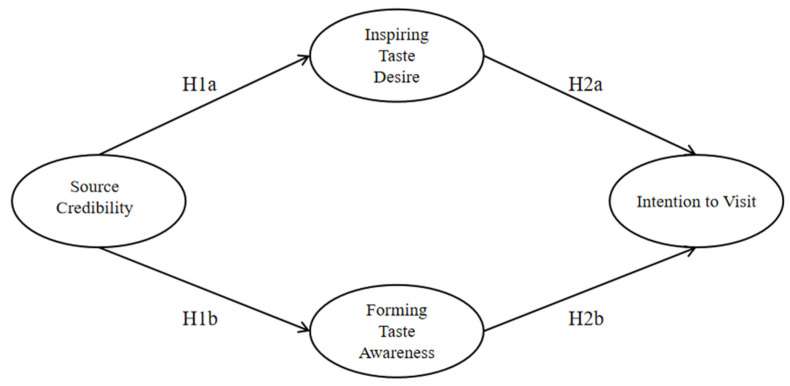
Conceptual framework of this study.

Based on the SOR theory, the conceptual framework of this study is shown in Figure 1, summarizing the relationships between Source Credibility, Insipiring Taste Desire, Forming Taste Awareness, and Intention to Visit.

## 3. Methodology

### 3.1. Data Collection

This study conducted a cross-sectional survey through questionnaires from October to November 2023. The questionnaire first explained the purpose of the research and the variables of interest. It included a screening question to confirm whether participants had watched food exploration videos on the Douyin platform. Researchers clarified the definition and characteristics of such videos in this question to ensure the sample matched the study’s requirements. Subsequently, participants were asked to evaluate based on their viewing experience of food exploration videos on Douyin and complete a series of questionnaires, including demographic information, a source credibility scale, scales for measuring inspiring taste desire and forming taste awareness, and an intention to visit scale. 

We commissioned a professional data survey agency, ‘Tencent Questionnaire’ (http://wj.qq.com, accessed on 14 November 2023.), to distribute online paid questionnaires (2500 CNY) for the specific data collection. The Tencent Questionnaire platform has an extensive sample database with more than 3 million potential respondents, covering a wide range of age groups, occupational backgrounds, and educational levels across most regions of China. The company was responsible for ensuring the quality and validity of the data during the questionnaire completion process. After the distribution of questionnaires and data collection, the company provided us with the final valid data. This study used the valid sample provided by the company and did not conduct additional screening. According to Hair and Alamer, we used G*Power 3.1.9.7 software to determine that the minimum sample size required for the structural equation model is 114 [70]. Based on previous similar studies [71,72] and considering potential data incompleteness and loss rates, we issued 500 questionnaires to ensure that even if some data were invalid, there would still be a sufficient sample size for practical statistical analysis. Subsequently, we cleaned the data thoroughly, excluding samples with missing values and other issues. After this process, we obtained 437 valid samples, corresponding to an effective response rate of 87.40%.

### 3.2. Measures

#### 3.2.1. Source Credibility

This study, drawing on the research of Munnukka et al. [39], asked respondents to indicate their level of agreement with a series of statements to measure perceived trustworthiness, expertise, attractiveness, and similarity. Each dimension was composed of 3 to 4 questions. Each statement began with ‘I think the food exploration blogger..’. and was rated using a 7-point Likert scale, ranging from ‘strongly disagree’ to ‘strongly agree’.

This study modified the wording of the questions to suit the cultural context better, thereby ensuring the questionnaire’s local adaptability. At the same time, concepts that could be easily confused were clearly explained and differentiated to enable participants to make more accurate judgments. For example, the trustworthiness measurement included questions like ‘I think the food exploration blogger is honest’. Expertise was measured with questions like ‘I think the food exploration blogger knows a lot about the food/places they share’. Attractiveness was assessed with questions like ‘I find the food exploration blogger physically attractive’. Similarity was measured with questions such as ‘I have much in common with the food exploration blogger’.

#### 3.2.2. Inspiring Taste Desire and Forming Taste Awareness

Wang first developed a structured questionnaire for measuring the factors influencing readers’ intentions regarding food blogs [26]. This study referred to the scales in that questionnaire for measuring the inspiring taste desire and the forming taste awareness variables. The inspiring taste desire variable has two dimensions: ‘Experiencing Appeal’ and ‘Generating Empathy’. The wording of some questions was modified in this study to better align with the theme of food exploration videos, and irrelevant questions were removed. Ultimately, the scale for inspiring taste desire comprised eight items. ‘Experiencing Appeal’ was measured with questions like ‘I think the content of the food exploration videos is exciting’. ‘Generating Empathy’ was assessed with questions like ‘I dream of tasting delicious food after watching food exploration videos’. The variable of forming taste awareness includes three dimensions: ‘Providing Image’, ‘Delivering Knowledge’, and ‘Presenting Guides’. This study modified the original questionnaire’s ‘food restaurants’ to ‘culinary locations’ to broaden the scope of culinary destinations, better matching the content of food exploration videos. Additionally, questions not fitting the Chinese regional culture, such as ‘I think the food blog helps me understand how to drink local wines’, were removed to make the questionnaire more relevant. The scale for forming taste awareness thus consisted of 12 items. ‘Providing Image’ was measured with questions like ‘I think food exploration videos let me know in advance that the culinary location offers fresh and healthy food’. ‘Delivering Knowledge’ was assessed with questions such as ‘I think food exploration videos let me understand the local traditions and customs of local cuisine’. ‘Presenting Guides’ was measured with questions like ‘I think food exploration videos offer suggestions on menu choices at culinary locations’. This section also used a 7-point Likert scale for measurement, with participants rating their agreement from ‘strongly disagree’ to ‘strongly agree’.

#### 3.2.3. Intention to Visit

The measurement of intention to visit also employed the scale developed by Wang [26]. This scale includes three items, reflecting the participants’ perceived attitudes: ‘planning’, ‘deciding’, and ‘tendency’. Each item assessing these attitudes utilized a 7-point Likert scale, ranging from ‘strongly disagree’ to ‘strongly agree’.

### 3.3. Analysis

This study conducted data analysis using SPSS 27.0 and AMOS 23.0 software. Initially, descriptive statistical analysis and bivariate relationship analysis were performed in SPSS 27.0 to obtain primary data characteristics and preliminary relationships between variables. Subsequently, Cronbach’s alpha was calculated within SPSS 27.0 to conduct a reliability test, assessing the internal consistency of the questionnaire. Following this, confirmatory factor analysis (CFA) and the calculation of average variance extracted (AVE) were conducted using AMOS to verify the theoretical consistency of the questionnaire’s factor structure and the convergent validity of the constructs. The structural equation model (SEM) fit was also tested to ensure a good match between the model and the data. Finally, the hypothesized path relationships within the model were examined in detail in AMOS to validate the hypotheses of this study.

## 4. Results

### 4.1. Preliminary Analysis

This study included a total of 437 participants. Regarding gender distribution, females accounted for 71.90%, totaling 314 individuals, while males accounted for 28.10%, totaling 123 individuals. The average age of participants was 24.13 years, with a standard deviation of 5.11 years. Regarding educational level, the highest qualification was a master’s degree or above, held by 5.70% or 25 participants, while the lowest was elementary education only, held by 4.10% or 18 participants (Table 1). Descriptive statistical analysis showed that the average scores for each variable ranged from 4 to 6, indicating that the target group generally ranks at a medium-or-above level regarding their cognition and behavior (Table 2). Additionally, the reliability and validity of the data were assessed, including Cronbach’s alpha and average variance extracted (AVE), to ensure that the measurement tools used possessed good internal consistency and construct validity (Table 3). Finally, the analysis of bivariate relationships revealed significant pairwise associations among all variables (Table 4).

### 4.2. Model Testing

**Figure 2:**
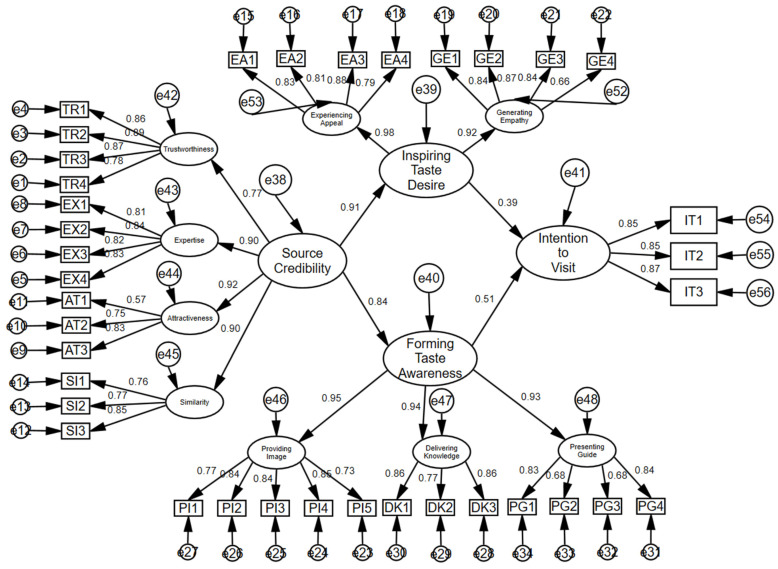
Path coefficients of hypothesized relations.

This study further explored the relationships between variables by conducting a structural equation modeling (SEM) analysis using AMOS 23.0 software and establishing a structural model diagram, as shown in Figure 2. Before this, confirmatory factor analysis (CFA) and fit tests of the SEM were carried out to ensure a good match between the model structure and the collected data. The CMIN/DF in the CFA was 2.34, and the RMSEA was 0.06. These values are within the ideal range Kline suggested, indicating that the data support the scale structure [73].

Additionally, the IFI, TLI, and CFI exceeded 0.9, demonstrating excellent model fit. After confirming the good fit of the CFA model, further fit tests were conducted using SEM for the entire model. The results showed that the overall model’s CMIN/DF was 2.62, with an RMSEA of 0.06, and the IFI, TLI, and CFI indices also exceeded 0.9. These indicators meet the standards Kline set, confirming the good overall fit of the model [73]. The firm fit of this model further supports the hypothesized path relationships among the measured variables, ensuring consistency between the theoretical structure of the model and the actual data.

Given the good fit of the model, we further conducted a detailed examination of the hypothesized path relationships within the model using AMOS software. According to the results of the path hypothesis relationship test (Table 5), source credibility significantly positively predicts the inspiring taste desire variable (β = 0.91, *p* < 0.001), thereby supporting hypothesis H1a. Specifically, trustworthiness shows a significant positive correlation with experiencing appeal (r = 0.60, *p* < 0.01) and generating empathy (r = 0.52, *p* < 0.01), indicating that viewers with higher levels of trustworthiness are more likely to experience intense taste desires. The high correlation between expertise and experiencing appeal (r = 0.70, *p* < 0.01) and generating empathy (r = 0.66, *p* < 0.01) suggests the importance of expertise in stimulating taste desires. Attractiveness also plays a crucial role in stimulating taste desires, showing significant correlations with experiencing appeal (r = 0.67, *p* < 0.01) and generating empathy (r = 0.67, *p* < 0.01). Moreover, the significant positive correlation between similarity and experiencing appeal (r = 0.71, *p* < 0.01) and generating empathy (r = 0.67, *p* < 0.01) indicates that viewers’ perception of similarity to the blogger significantly impacts stimulating gustatory resonance. 

Source credibility also significantly positively predicts the forming taste awareness variable (β = 0.84, *p* < 0.001), thus confirming hypothesis H1b. Trustworthiness, expertise, attractiveness, and similarity strongly correlate with the various dimensions of forming taste awareness. Specifically, the correlations include trustworthiness with providing image (r = 0.59, *p* < 0.01), delivering knowledge (r = 0.46, *p* < 0.01), and presenting guides (r = 0.45, *p* < 0.01); expertise with providing image (r = 0.70, *p* < 0.01), delivering knowledge (r = 0.57, *p* < 0.01), and presenting guides (r = 0.56, *p* < 0.01); attractiveness with providing image (r = 0.61, *p* < 0.01), delivering knowledge (r = 0.56, *p* < 0.01), and presenting guides (r = 0.56, *p* < 0.01); and similarity with providing image (r = 0.63, *p* < 0.01), delivering knowledge (r = 0.56, *p* < 0.01), and presenting guides (r = 0.54, *p* < 0.01). These results indicate that trustworthiness, expertise, attractiveness, and similarity are crucial in shaping the audience’s imagination and understanding of culinary locations by providing rich, relatable content that resonates with viewers.

Furthermore, inspiring taste desire significantly positively predicts the intention to visit (β = 0.39, *p* < 0.001), supporting hypothesis H2a. Experiencing appeal (r = 0.72, *p* < 0.01) and generating empathy (r = 0.70, *p* < 0.01) towards the food exploration videos show a strong positive correlation with the intention to visit. Forming taste awareness also has a significant positive predictive effect on the intention to visit (β = 0.51, *p* < 0.001), validating hypothesis H2b. The significant positive correlations between dimensions such as providing image, delivering knowledge, and presenting guides further confirm this, with the correlation between presenting guides and intention to visit being the highest (r = 0.72, *p* < 0.01), highlighting the importance of practical information in stimulating the intention to visit.

## 5. Discussion

Based on the SOR model, this study explored the impact of Douyin food exploration videos on consumer behavior. The analysis results revealed that source credibility (S) has a significant positive predictive effect on inspiring taste desire (O) and forming taste awareness (O). It indicates that the blogger’s trustworthiness, expertise, attractiveness, and similarity are closely linked to consumers’ cognition and behavioral responses to culinary locations. These findings highlight the critical role of source credibility in consumer decision-making, particularly in stimulating consumers’ gustatory desires and forming specific imaginations about culinary destinations.

### 5.1. The Influence of Information Sources: From Blogger Traits to Consumer Perception

This study delves into the role of information sources in influencing consumer perception, yielding the following findings: First, source credibility significantly positively predicts both inspiring taste desire and forming taste awareness. It indicates that bloggers not only arouse viewers’ curiosity and desire to explore culinary locations but also help them form a comprehensive understanding of these places in advance. Second, identity recognition plays a role in enhancing the attractiveness of the culinary experience for viewers. The results show that similarity has the strongest correlation with gustatory desires among the four characteristics of influencers. It may partly be because food exploration bloggers often reveal their taste preferences in their videos. Viewers may develop a stronger desire for the food experiences described when they perceive similarities in food preferences with the blogger. Additionally, if viewers identify with the personality traits or life attitudes presented by the blogger, it can trigger a more profound sense of identification. This identity-based identification may lead viewers to imitate the blogger’s behavior [74]. Third, the blogger’s expertise is essential in forming the viewer’s gustatory awareness, consistent with existing research findings. Xu and Huang suggest that information plays a crucial role in shaping consumers’ cognitive expectations about restaurants and food [75]. The professionalism exhibited by bloggers increases viewers’ trust in the reliability of their information [76]. On the Douyin platform, expertise becomes a distinguishing factor among many food exploration bloggers. By showcasing their professional knowledge and experiences, bloggers enhance viewers’ trust in the reliability of their information. This professional-level information transfer enables viewers to mentally pre-experience and imagine the taste, texture, and enjoyment of the food, as well as gain a preliminary understanding of the culinary location’s environment, thereby promoting the formation of gustatory awareness.

### 5.2. The Driving Role of Internal Psychological States on Behavioral Responses

In the organism–response (O-R) link, we found that both inspiring taste desire and forming taste awareness significantly predict the intention to visit. It indicates that the viewers’ gustatory desires for food are critical drivers in motivating them to form the intention to visit specific dining locations. When viewers experience a strong sensory attraction to food through the content of food exploration bloggers, this sensory stimulus is transformed into a strong desire to experience these foods [77]. It confirms the crucial role of internal psychological states in driving consumer behavioral responses. In other words, as an intrinsic motivational response, gustatory desire appears to be closely linked with interest in culinary locations and an enhanced intention to visit. Additionally, consistent with the research of Chen et al. [78], if food blogs effectively help readers build a clear and comprehensive cognitive impression of a culinary location, their role in enhancing their intention to visit that place is significantly strengthened.

### 5.3. The Impact of Practical Information and Delivering Knowledge on Decision-Making

This study explored the impact of different concepts on behavioral intentions in the cognitive-to-behavioral conversion pathway. While all the concepts similarly relate to behavioral intentions, the most noteworthy association is with guiding information. According to the theory of bounded rationality [79], people face limitations in information processing when making decisions, and clear options and practical advice can help alleviate this burden, thus making the decision-making process more efficient. Therefore, when viewers watch food exploration videos that provide rich guidance, they are more inclined to feel confident and prepared to visit these recommended places, indicating that viewers value practical and helpful information more.

Contrary to the findings of Wang [26], our study discovered that the transfer of knowledge has a significant impact on forming the intention to taste. It may be closely linked to the richness and diversity of Chinese culinary culture. Local specialty cuisines are often a crucial factor in deciding travel destinations [80,81], especially in China, where regional culinary characteristics are unique and diverse. Therefore, imparting knowledge about local food cultures helps audiences understand these cultural features and stimulates their willingness to experience these cultures in person. At the same time, understanding the dining etiquette and cooking methods of different regions in advance can help reduce the uncertainty associated with unfamiliar foods, enhancing the desire to taste these cuisines.

## 6. Implications

On a theoretical level, first, this study applied and validated the stimulus–organism–response (SOR) model in the context of food exploration videos on the Douyin platform. It enhances the understanding of the applicability of the SOR model in different fields and contexts. Secondly, this study expanded the understanding of the role of source characteristics (such as the trustworthiness, expertise, attractiveness, and similarity of bloggers) in forming consumer perceptions. Particularly in the context of social media platforms like Douyin, these traits influence consumers’ gustatory desires and willingness to taste. Lastly, by exploring identity alignment in the relationship between consumers and content creators, this study provides a new perspective for understanding the dynamics between social media influence and consumer behavior.

On a practical level, firstly, the research findings provide strategic guidance for food exploration bloggers and other content creators, such as emphasizing expertise and establishing identity alignment with the audience to enhance the appeal and impact of their content. Secondly, this study highlights the importance of practical information (such as providing guides) in influencing consumer decisions, which is crucial for marketers and advertisers in designing promotional strategies. Finally, for social media platforms (such as Douyin), understanding which blogger characteristics appeal to the audience can help platforms manage and promote content more effectively, attracting and retaining a broader audience.

## 7. Limitations and Future Research

This study primarily collected data through a survey. While this method effectively gathers a large sample, it may not deeply capture participants’ thoughts and motivations, particularly regarding consumer perceptions and behavioral intentions. Future research will employ qualitative methods such as in-depth interviews to understand the thought patterns and motivational drivers behind consumer behavior. Moreover, this study did not fully consider other environmental factors, such as online reviews, which could significantly impact consumer perceptions and behaviors. Future research will consider incorporating online reviews and other social media content into the analytical framework to explore their effects on consumer perceptions and actions. There are various types of food exploration bloggers on Douyin, and different types of bloggers may influence the audience in various ways. However, this study did not differentiate between types of bloggers. Future research will investigate the impact of different types of bloggers on audience cognition and behavior to more accurately understand the influence of various content creators. Regarding participants, this study did not involve their socioeconomic characteristics. Future research could consider incorporating these features to enrich the depth and breadth of the research findings. Additionally, this study focused on the Douyin platform, while other social media platforms also host numerous food bloggers. Future studies might consider comparing the role of food exploration bloggers across different social platforms to explore how various online environments specifically affect consumer perceptions and behaviors. Lastly, we hope that future research will expand the scope of the literature review further to enhance this study’s international perspective and cultural diversity and to understand the influence of social media globally more comprehensively.

## 8. Conclusions

This study utilized the SOR model to explore how the characteristics of food exploration bloggers on the Douyin platform influence audience perception and behavioral intentions. By analyzing data from 437 participants, the research revealed the significant positive role of food exploration bloggers as a source of information in stimulating the audience’s taste desires and cognition of food locations, which further influenced their intention to visit. The findings of this study highlight the potential of applying the SOR model in a social media context, providing new insights into understanding the impact of different types of information sources on audience responses. These discoveries enhance the applicability of the SOR model in digital media research.

## Figures and Tables

**Table 1:** Demographic characteristics of participants (N = 437).

Category		Number/Average Age	Percentage/Standard Deviation
Gender	Female	314	71.90%
	Male	123	28.10%
Age	Average age	24.13 years	SD = 5.11
Education	Bachelor’s degree	222	50.80%
	Associate’s degree	111	25.40%
	High school/vocational school	61	14.00%
	Master’s degree and above	25	5.70%
	Elementary school	18	4.10%

**Table 2:** Dimension descriptive statistics.

Dimension	M	SD
Trustworthiness	4.49	1.07
Expertise	4.75	1.16
Attractiveness	4.75	1.12
Similarity	4.60	1.24
Experiencing appeal	5.01	1.16
Generating empathy	5.25	1.43
Providing image	5.19	1.10
Delivering knowledge	5.30	1.15
Presenting guides	5.29	1.06
Intention to visit	5.21	1.17

**Table 3:** Reliability and validity measures for study variables.

Variable	Number of Items	Cronbach’s Alpha	AVE
Trustworthiness	4	0.91	0.73
Expertise	4	0.90	0.68
Attractiveness	3	0.76	0.53
Similarity	3	0.84	0.63
Source credibility	14	0.94	0.77
Experiencing appeal	4	0.89	0.69
Generating empathy	4	0.87	0.65
Inspiring taste desire	8	0.93	0.90
Providing image	5	0.90	0.65
Delivering knowledge	3	0.87	0.69
Presenting guides	4	0.87	0.66
Forming taste awareness	12	0.95	0.88
Intention to taste	3	0.89	0.78

**Table 4:** Pearson correlation analysis results among various dimensions.

Dimension	Trustworthiness	Expertise	Attractiveness	Similarity	Experiencing Appeal	Generating Empathy	Providing Image	Delivering Knowledge	Presenting Guides	Intention to Visit
Trustworthiness	1									
Expertise	0.78 **									
Attractiveness	0.62 **	0.66 **								
Similarity	0.61 **	0.69 **	0.69 **							
Experiencing appeal	0.60 **	0.70 **	0.67 **	0.71 **						
Generating empathy	0.52 **	0.66 **	0.67 **	0.67 **	0.82 **					
Providing image	0.59 **	0.70 **	0.61 **	0.63 **	0.76 **	0.74 **				
Delivering knowledge	0.46 **	0.57 **	0.56 **	0.56 **	0.70 **	0.69 **	0.80 **			
Presenting guides	0.45 **	0.56 **	0.56 **	0.54 **	0.70 **	0.71 **	0.77 **	0.79 **		
Intention to visit	0.54 **	0.60 **	0.58 **	0.61 **	0.72 **	0.70 **	0.71 **	0.67 **	0.72 **	1

** At the level of 0.01 (two-tailed), the correlation is significant.

**Table 5:** Path relation test result.

Path Relation			Estimate	S.E.	C.R.	*p*
Source credibility	—>	Inspiring taste Desire	0.91	0.10	13.67	***
Source credibility	—>	forming Taste awareness	0.84	0.09	12.81	***
Inspiring taste Desire	—>	Intention to visit	0.39	0.06	6.47	***
forming Taste awareness	—>	Intention to visit	0.51	0.07	8.20	***

*** At the level of 0.001 (two-tailed), the path coefficient is highly significant.

## Data Availability

The data are available from the authors upon reasonable request.
